# Pannexin 1 dysregulation in Duchenne muscular dystrophy and its exacerbation of dystrophic features in *mdx* mice

**DOI:** 10.1186/s13395-024-00340-8

**Published:** 2024-04-26

**Authors:** Emily Freeman, Stéphanie Langlois, Marcos F. Leyba, Tarek Ammar, Zacharie Léger, Hugh J. McMillan, Jean-Marc Renaud, Bernard J. Jasmin, Kyle N. Cowan

**Affiliations:** 1 Department of Surgery, Division of Pediatric Surgery, University of Ottawa, Children’s Hospital of Eastern Ontario, Ottawa, ON Canada; 2 Children’s Hospital of Eastern Ontario Research Institute, Pediatric General Surgery, 401 Smyth Rd, Room 3360, Ottawa, ON K1H 8L1 Canada; 3 Department of Cellular and Molecular Medicine, University of Ottawa, Ottawa, ON Canada; 4 Centre for Neuromuscular Disease, Ottawa, ON Canada

**Keywords:** Duchenne muscular dystrophy, Myoblast, Myofiber, Pannexin 1, Skeletal muscle

## Abstract

**Background:**

Duchenne muscular dystrophy (DMD) is associated with impaired muscle regeneration, progressive muscle weakness, damage, and wasting. While the cause of DMD is an X-linked loss of function mutation in the gene encoding dystrophin, the exact mechanisms that perpetuate the disease progression are unknown. Our laboratory has demonstrated that pannexin 1 (Panx1 in rodents; PANX1 in humans) is critical for the development, strength, and regeneration of male skeletal muscle. In normal skeletal muscle, Panx1 is part of a multiprotein complex with dystrophin. We and others have previously shown that Panx1 levels and channel activity are dysregulated in various mouse models of DMD.

**Methods:**

We utilized myoblast cell lines derived from DMD patients to assess PANX1 expression and function. To investigate how Panx1 dysregulation contributes to DMD, we generated a dystrophic (*mdx*) mouse model that lacks Panx1 (*Panx1*^*−/−*^*/mdx*). In depth characterization of this model included histological analysis, as well as locomotor, and physiological tests such as muscle force and grip strength assessments.

**Results:**

Here, we demonstrate that PANX1 levels and channel function are reduced in patient-derived DMD myoblast cell lines. *Panx1*^*−/−*^*/mdx* mice have a significantly reduced lifespan, and decreased body weight due to lean mass loss. Their *tibialis anterior* were more affected than their *soleus* muscles and displayed reduced mass, myofiber loss, increased centrally nucleated myofibers, and a lower number of muscle stem cells compared to that of *Panx1*^+*/*+^*/mdx* mice. These detrimental effects were associated with muscle and locomotor functional impairments. In vitro, PANX1 overexpression in patient-derived DMD myoblasts improved their differentiation and fusion.

**Conclusions:**

Collectively, our findings suggest that PANX1/Panx1 dysregulation in DMD exacerbates several aspects of the disease. Moreover, our results suggest a potential therapeutic benefit to increasing PANX1 levels in dystrophic muscles.

**Supplementary Information:**

The online version contains supplementary material available at 10.1186/s13395-024-00340-8.

## Background

Duchenne muscular dystrophy (DMD), the most common childhood-onset neuromuscular disorder, affects roughly 1 in 3600 to 1 in 6000 male live births [6]. DMD is caused by an X-linked loss of function mutation in the gene encoding dystrophin, leading to a lack of the functional protein [6, 23]. In the less severe Becker muscular dystrophy (BMD), decreased expression of dystrophin leads to an attenuated form of the disease. Loss of dystrophin results in contraction-induced myofiber damage. During the early stages of the disease, degenerating muscles undergo repetitive cycles of regeneration. As satellite cells (SCs) are depleted and eventually exhausted, extensive structural damage, progressive muscle wasting, adipocyte infiltration, inflammation, eventual paralysis, and respiratory or cardiac failure ensues [10, 15, 26, 27]. Unfortunately, there is no available cure for DMD and most patients will die from cardiorespiratory complications in the second or third decade of life [6, 27]. While the cause of DMD is well established, the specific factors that potentiate disease progression are not well understood, complicating the search for effective therapeutics.

Pannexin 1 (Panx1 in rodents; PANX1 in humans) forms single membrane channels permeable to molecules > 1 kDa in size such as ATP [33]. We have previously shown that pannexin 1 plays a key role in myogenesis in humans and mice [19, 28]. In addition to differentiated skeletal muscle fibers, PANX1/Panx1 is expressed in SCs and myoblasts [19, 36, 42]. PANX1/Panx1 levels increase during myogenesis in vitro, as well as in muscle development and regeneration in vivo [28, 36]. In differentiated fibers, the enhancement of contractile force is dependent upon Panx1-mediated ATP release [38]. A role for Panx1 in regulating skeletal muscle regeneration by promoting bleb-based myoblast migration and fusion was also demonstrated [42]. Furthermore, we recently showed that the role Panx1 holds in regulating skeletal muscle development and regeneration is sex dependent. Specifically, Panx1 plays a significant function in regulating muscle strength, as well as muscle fiber size during development and regeneration in male, but not in female mice. Panx1 is also required for the development of the SC pool and myoblast fusion only in male mice [19].

Cea et al. have previously shown that Panx1 levels are increased in the *tibialis anterior* (TA) muscle of 2–3-month-old dystrophic *mdx*^*4cv*^ (mouse model of DMD) mice compared to their controls [9]. Myofibers from *flexor digitorum brevis* muscles isolated from these mice displayed enhanced Panx1 channel activity based on ethidium uptake [9]. Valladares et al. found elevated levels of Panx1 in triad fractions from *mdx* muscles compared to those from C57BL/6 mice [44]. ATP released through Panx1 channels after electrical stimulation activates signaling pathways regulating the expression of genes that define the fast-to-slow fiber phenotype transition [7, 24]. In normal skeletal muscle, Panx1 co-immunoprecipitates with dystrophin as part of a multiprotein complex involved in this excitation-transcription coupling [3, 44]. The excitation-induced Panx1-mediated ATP signaling that regulates myogenic gene expression may thus be disrupted in DMD due to the absence of dystrophin. Indeed, the basal levels of extracellular ATP were higher in *mdx* fibers than in normal fibers but *mdx* fibers were unable to activate Panx1-mediated ATP release after electrical stimulation [44]. This may be involved in increased expression of cell death genes in *mdx* fibers that could result in muscle cell loss in dystrophy. Moreover, we have previously reported that Panx1 protein levels were significantly decreased in muscles from a severe mouse model of DMD (*utrn*^*−/−*^*/mdx*) [36]. Collectively, these data suggest that Panx1 levels and function are altered in various models of DMD however, the role of Panx1 in DMD and its progression remained to be investigated.

Here we demonstrate that despite varying PANX1 protein levels in patient-derived DMD myoblast cell lines, the mean PANX1 level in myoblasts from DMD patients was less than that of healthy donors. Furthermore, myoblasts from dystrophic patients displayed reduced PANX1 channel function. To assess the role of Panx1 in DMD, we generated a dystrophic mouse line that lacks *Panx1* (*Panx1*^*−/−*^*/mdx*). Notably, the genetic ablation of *Panx1* significantly reduced the lifespan of *mdx* mice. These findings were associated with a smaller body weight attributed to loss of lean mass. The *tibialis anterior* (TA) and to a lesser degree the *soleus* muscles of *Panx1*^*−/−*^*/mdx* mice showed altered fiber size distribution and an increased proportion of myofibers with centrally located nuclei. Their TA muscle also had a reduced wet mass and contained fewer myofibers and SCs. Moreover, *Panx1*^*−/−*^*/mdx* mice displayed reduced muscle force and locomotor function. In vitro, the overexpression of PANX1 in patient-derived DMD myoblasts enhanced their differentiation and fusion capacities. Taken together, this study demonstrates that PANX1 channel activity is reduced in muscle cells from DMD patients and shows that *Panx1* loss in *mdx* mice exacerbates several aspects of dystrophy in vivo. While suggesting that Panx1 holds a protective role in dystrophic fibers, our data also imply that increasing Panx1 levels may be beneficial to promote muscle formation/regeneration of dystrophic fibers.

## Materials and methods

### Tissue specimens and patient-derived cell lines

After institutional ethics board approval (CHEO Research Ethics Board, protocol CHEOREB# 19/49X), archived frozen pediatric muscle samples were obtained, as secondary use of clinical samples, from the Department of Pathology and Laboratory Medicine, Children’s Hospital of Eastern Ontario (CHEO), Ottawa, Ontario, Canada. Experiments were carried out in accordance with the CHEO Ethics Board guidelines and regulations.

Immortalized human skeletal muscle myoblasts (HSMM) generated from biopsies obtained from 8 DMD patients and 3 controls were acquired from Dr. Bénédicte Chazaud and previously described in Massenet et al. [31]. HSMM were cultured in Ham’s F14 media supplemented with 30% FBS, 10 µg/ml of insulin (16634, Sigma-Aldrich, St. Louis MO, USA), 25 ng/ml FGF-2 (SRP4037, Sigma-Aldrich), 10 ng/ml hEGF (E9644, Sigma-Aldrich), 2 µg/ml amphotericin B (A2942, Sigma-Aldrich), and 1% penicillin/streptomycin at 37 °C, 5% CO_2_.

### Transfection

Human skeletal muscle myoblasts (HSMM) on collagen-coated 35 mm dishes were transfected with 5 nM of Silencer® Select siRNA targeting *PANX1* (5’ CGAUCAGUUUCAGUGCAAAtt 3’) or 5 nM of the Silencer® Select Negative Control No. 1 (ambion 4390843, Thermo Fisher Scientific, Carlsbad CA, USA) using Lipofectamine 2000 (Thermo Fisher Scientific) as per the manufacturer’s protocol. Dye uptake assays were performed 48 h post-transfection. Once completed, cell lysates were collected to confirm successful knockdown via western blotting.

### Dye uptake assays

The low [K^+^] solution (145 mM NaCl, 5 mM KCl, 1.4 mM CaCl_2_, 1 mM MgCl_2_, 10 mM HEPES diluted in ddH_2_O) and high [K^+^] solution (60 mM NaCl, 50 mM KCl, 1.4 mM CaCl_2_, 1 mM MgCl_2_, 10 mM HEPES diluted in ddH_2_O) were prepared as described by [41]. Cells were washed twice with either the low [K^+^] or high [K^+^] solution, then incubated in fresh low [K^+^] or high [K^+^] solution for 5 min at 37 °C, in 5% CO_2_. Next, cells were incubated with 4 mg/ml of sulforhodamine B dye (S1307, Invitrogen, Waltham MA, USA) dissolved in low [K^+^] or high [K^+^] solution for 15 min at 37 °C, in 5% CO_2_, washed, and imaged immediately with the EVOS FL Auto fluorescent microscope (Thermo Fisher Scientific) under 10X magnification. Ten random fields of view were imaged per slide. Images were counted for the incidence of dye uptake defined as the percentage of cells that took up the dye.

### Experimental animals

All experiments were conducted in accordance with the University of Ottawa Animal Care Guidelines and the Canadian Council of Animal Care Guidelines. Mice were housed under a 12 h light/dark cycle with food and water ad libitum. *Mdx* (C57BL/10ScSn-Dmd^mdx^/J) mice were purchased from The Jackson Laboratory (strain #001801). *Panx1*^*−/−*^ mice were generated by Dr. Valery Shestopalov [16] and back-crossed onto C57BL/6J for another six times in the Dr. Leigh Anne Swayne laboratory [39]. To generate *mdx* mice lacking *Panx1*, male *Panx1*^*−/−*^ mice were crossed with female *mdx* (*DMD*^*mdx/mdx*^) mice to generate *Panx1*^+/-^*/DMD*^*mdx/Y*^ male mice. Male *Panx1*^+/-^*/DMD*^*mdx/Y*^ mice were then crossed with female *mdx* mice. Resulting *Panx1*^+/-^*/DMD*^*mdx/Y*^ male mice and *Panx1*^+/-^*/DMD*^*mdx/mdx*^ female mice were crossed to generate the experimental male *Panx1*^*−/−*^*/mdx* (*Panx1*^*−/−*^*/ DMD*^*mdx/Y*^) mice and their control *Panx1*^+*/*+^*/mdx* (*Panx1*^+*/*+^*/ DMD*^*mdx/Y*^) mice. Genotypes were determined by PCR screening of ear notch DNA for the *Panx1* deletion as described by Sanchez-Arias et al. [39] and for the nonsense mutation in exon 23 of the *dystrophin* gene as described by Shin et al. [40]. All measurements and assessments were done in a blinded fashion. Body weights were recorded as well as survival.

### Tissue processing

Male (12‐week‐old) mice were euthanized and their *tibialis anterior* (TA) and *soleus* (Sol) muscles were harvested. Muscles were either embedded in OCT compound (Thermo Fisher Scientific) by freezing in isopentane pre-cooled in liquid nitrogen or fixed in 4% paraformaldehyde for 24 h at room temperature then stored in 70% ethanol until paraffin embedding. Cross-sectional area (CSA), fiber number, and central nuclei analysis were completed in frozen muscle sections stained with Hematoxylin and Eosin, while Masson trichrome staining was done on paraffin embedded sections. Paraffin embedding, tissue processing, and staining of paraffin-embedded tissue were performed at the Louise Pelletier Histology Core Facility at the University of Ottawa.

### Hematoxylin & eosin, masson trichrome, and oil red o staining

Hematoxylin and Eosin (H&E) (Sigma‐Aldrich) staining was performed on muscle sections as described in [19]. The Masson trichrome and Oil Red O staining was done according to manufacturer’s protocols at the Louise Pelletier Histology Core Facility. Entire tissue sections were scanned using the EVOS FL Auto (Thermo Fisher Scientific) under 10X magnification. CSA was measured from randomly selected fibers (100–250 fibers for the Sol and 200–500 for the TA). Fiber number counts were performed on the entire section and represented as fibers per mm^2^. Central nuclei counts were performed on entire muscle scans and data represents the average of three serial sections. For fat infiltration, data represents the percentage of area stained in red by Oil Red O. For the Masson trichrome staining, data represents the percentage of muscle section area stained in blue (collagen) [45]. All measurements were calculated using the ImageJ (FIJI) software.

### Maximum force and force frequency curve

Control solution contained (in mM): 118.5 NaCl, 4.7 KCl, 2.4 CaCl_2_, 3.1 MgCl_2_, 25 NaHCO_3_, 2 NaH_2_PO_4_, and 5.5 d-glucose. Solutions were continuously bubbled with 95% O_2_ – 5% CO_2_ to maintain a pH of 7.4. Experiments were performed at room temperature. Total flow of solutions in the muscle chamber was 15 ml/min being split just above and below the muscle to prevent any buildup of reactive oxygen species.

Mice (12-week-old) were euthanized, and their *soleus* were then dissected out. Muscle length was adjusted to give maximal tetanic force. Muscles were positioned horizontally in a Plexiglas chamber. One end of the muscle was fixed to a stationary hook, whereas the other end was attached to a force transducer (model400A; Aurora Scientific Canada). Similarly to methods described in Ammar et al. [2] the transducer was connected to a data acquisition system (KCP13104,Keithley), and data were recorded at 5 kHz [2]. Electrical stimulations were applied across two platinum wires (4 mm apart) located on opposite sides of the fibers. They were connected to a Grass S88 stimulator and a Grass SIU5 isolation unit (Grass Technologies, Cheshire UK). Tetanic contractions were elicited with 200-ms trains of 0.3-ms, 10-V (supramaximal voltage) pulses. Stimulation frequencies were set to give maximum tetanic force (140 Hz during maximization and equilibrium stages.) The force–frequency relationship was measured after a 30 min equilibrium period over a range from 1- 200 Hz. Twitch and tetanic force, defined as the force developed following a single stimulation pulse or a train of pulses, respectively, was calculated as the difference between the maximum force during a contraction and the force measured 5 ms before the contraction was elicited. Forces are presented in N/cm^2^.

### EchoMRI, forelimb grip strength, and pole descending test

EchoMRI was performed by a technician at the Animal Behavior Core Facility at the University of Ottawa on the EchoMRI-700 body composition analyzer and associated software (Houston, TX, USA).

Forelimb grip strength measurements of 12‐week‐old mice were completed using the Chatillon DFE II digital meter (Columbus Instruments, Columbus, USA), as we have previously described [19]. Following acclimatization to the workspace, each mouse was allowed to grip the grid firmly and then gently pulled horizontally relative to the grid until release. The maximum peak force value was recorded. This was repeated for a total of five times for each mouse with 10–15 s intervals between trials.

For the pole descending test, 12-week-old mice were placed near the top of a textured metal pole (diameter: ~ 8 mm; height: ~ 55 cm) with their nose facing upwards. The time taken to turn completely around (time to turn) and to climb down the pole face down were recorded (time to descend). If the animal fell from the pole, the maximum value of 120 s was given. Mice were tested in 5 consecutive trials. [21]. Grip strength measurements and the pole test were also performed at the Animal Behavior Core Facility at the University of Ottawa.

### Differentiation assays

For the differentiation assays, HSMM (70 000 cells/well) were seeded in 24-well dishes containing one collagen coated glass cover slip. Cells were transduced with a lentivector containing either GFP or PANX1 [46] one day prior to induction of differentiation. Based on GFP, a transduction efficiency of approximately 50% was achieved. Differentiation was initiated by switching cells into differentiation medium which consisted of DMEM supplemented with 5% horse serum and 1% penicillin/streptomycin. Cells were fixed in 3.7% PFA on day 5 of differentiation. Cells were stained for myosin heavy chain (MHC) (1:500, MF20, MAB4470, R&D Biosystems, Minneapolis, MN, USA), and mounted with Fluoromount-DAPI (Southern Biotech, Birmingham, AL, USA). Cells were imaged with the EVOS FL Auto fluorescent microscope (Thermo Fisher Scientific) under 20X magnification. The differentiation index (number of MHC positive cells/ total number of nuclei (%)) and fusion index (number of nuclei in myotubes/total number of nuclei (%)) were analyzed from 3–5 randomly selected fields of view [34].

### Immunofluorescence

Tissue and myoblasts were fixed in 3.7% PFA diluted in PBS for 20 min and permeabilized in 0.1 M glycine and 0.1% Triton X-100 in PBS for 10–15 min. Non-specific labeling was blocked for 1 h in 5% horse serum, 2% BSA and 0.1% Triton X-100 in PBS. Samples were incubated with antibodies against PANX1 (1:200, HPA016930 Sigma, St. Louis, MO, USA), Pax7 (1:2, hybridoma cell supernatant, DSHB, Iowa city, IA), MHC (1:500, MF20, cat# MAB4470, R&D Biosystems, Minneapolis, MN, USA), laminin (1:250, ab11575 Abcam, Cambridge, UK) at either room temperature for 1 h. Incubation with anti-rabbit Alexa Fluor 488, anti-mouse Alexa Fluor 488 or anti-rabbit Alexa Fluor 594, anti-mouse Alexa Fluor 594 (1:500–1:1000, A11008, A11017, A11005 and A11012, Life technologies, Eugene, OR, USA) antibodies were done at room temperature for 1 h. Myoblasts nuclei were stained with Hoescht 33,342 (Invitrogen). Tissue specimens were mounted with Fluoromount-DAPI (Southern Biotech, Birmingham, AL, USA). Samples were visualized with either the EVOS FL Auto (ThermoScientific) under 20X magnification or with the Olympus Fluoview FV1000 confocal microscope under either 20X or 60X magnification.

### Western blotting

Cells were lysed in 150 mM NaCl, 10 mM Tris, 1 mM EDTA, 1 mM EGTA, 1% Triton, 0.5% NP-40 and protease/phosphatase inhibitors (5872S, Cell Signaling, Danvers, MA, USA) for 1 h on ice then centrifuged at 12 000 rpm to remove cell debris. Equal amounts of protein were separated by SDS-PAGE, transferred to PVDF membranes, and non-specific binding was blocked with 5% bovine serum albumin in PBS + 0.01% Tween 20. Membranes were incubated with anti-PANX1 (1:1000, HPA016930, Sigma),anti-β-actin (1:5000, 8H10D10, Cell Signaling), or anti-GAPDH (1:5000; 14C10; Cell Signaling) followed by Alexa 680- (1:5000, A21009, Thermo Fisher Scientific, Carlsbad CA, USA) or infrared fluorescent-labeled secondary antibodies IRDye800 (1:5000, 925–32210, Li-COR Biosciences, Lincoln, NE, USA). Immunoblots were scanned with the Odyssey infrared-imaging system (LI-COR biosciences) and quantified using the ImageJ software.

### Statistics

The data were analyzed using unpaired or paired two-tailed student’s *t*-tests, one-way or two-way ANOVA followed by Tukey’s or Sidak’s post hoc tests as specified in the figure legends. All data are represented as mean ± s.d. unless otherwise stated. The number of animals used for each experiment is indicated in the figure legends. The individual data (each animal) points are also displayed on the graphs.

## Results

### PANX1 level and channel function are reduced in immortalized myoblasts from DMD patients

**Fig. 1:**
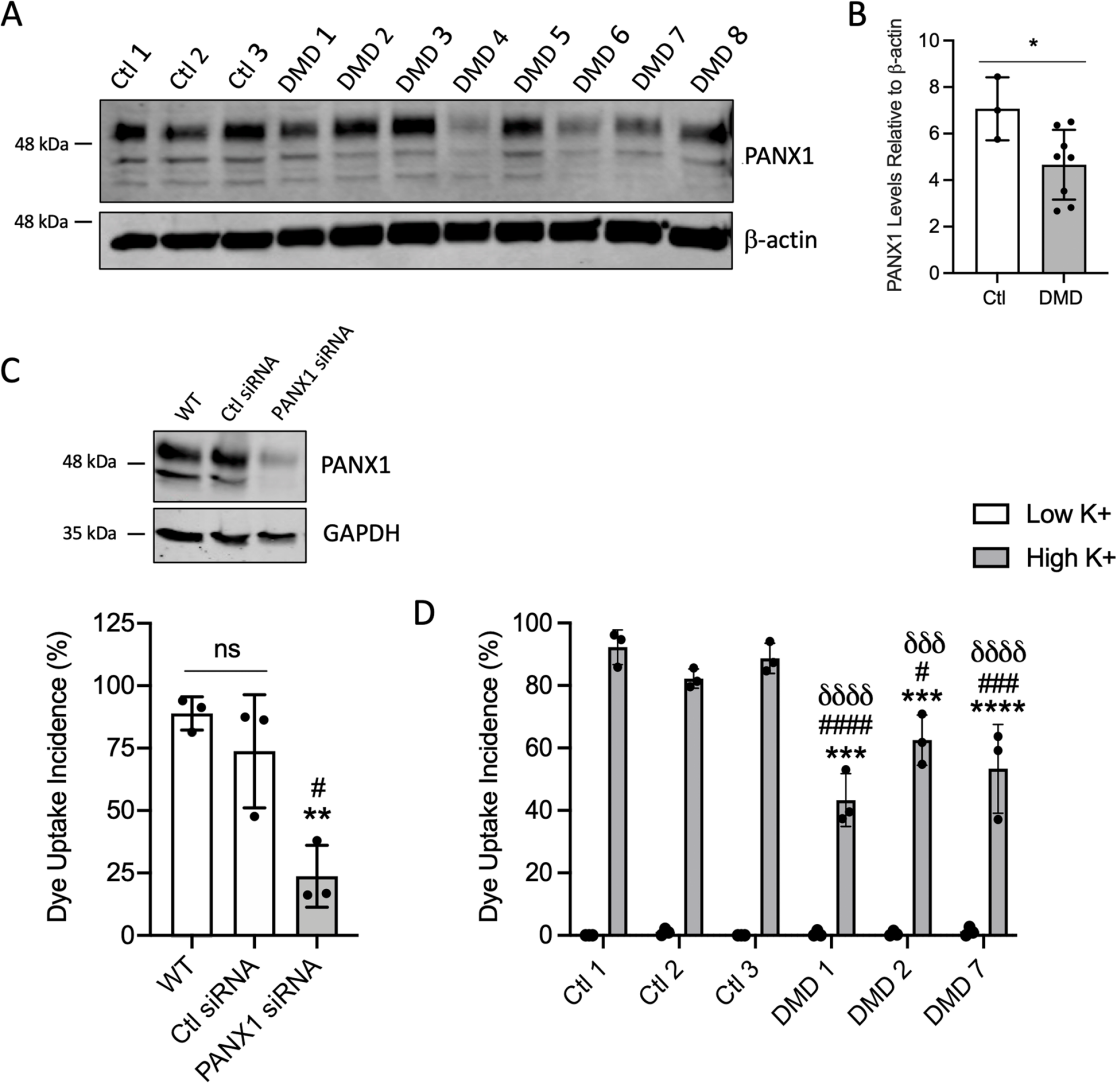
PANX1 Level and Channel Function are Reduced in Immortalized Myoblasts from DMD Patients. **A** Western blots of PANX1 levels in immortalized human skeletal muscle myoblasts isolated from 3 healthy (Ctl) and 8 dystrophic (DMD) donors and **B**) the respective quantification. β-actin was used as a loading control. * *P* < 0.05 compared to Ctl. **C** Representative western blot of Ctl myoblasts that were treated with either siRNA targeting *PANX1* (*PANX1* siRNA), a scrambled control (Ctl siRNA) or were untreated (WT: wild-type) (top). The incidence of dye uptake (%) in WT, Ctl siRNA, and *PANX1* siRNA cells in high [K^+^] (bottom) (*n* = 3; one-way ANOVA followed by Tukey’s multiple comparisons test). Data represents mean ± s.d. ** *P* < 0.01 in comparison to WT; # *P* < 0.05 in comparison to Ctl siRNA; ns: non-significant. **D** Incidence of dye uptake in cell lines Ctl1-3 and DMD 1, DMD 2, and DMD 7 in the presence of either low [K^+^] or high [K^+^] (*n* = 3; two-way ANOVA followed by Sidak’s multiple comparisons test). Data are represented as mean ± s.d. While not indicated on the graph, the difference between low [K^+^] and high [K^+^] was statistically significant for all cell lines (*P* < 0.0001). There was no statistical difference between the various cell lines in low [K^+^]. The statistical significance between the Ctl and DMD cell lines in high [K^+^] are indicated on the graph: *** *P* < 0.001 and **** *P* < 0.0001 in comparison to Ctl 1; # *P* < 0.05, ### *P* < 0.001, #### *P* < 0.0001 in comparison to Ctl 2; ððð *P* < 0.001 and ðððð *P* < 0.0001 in comparison to Ctl 3 in high [K.^+^]

To gain insight into how PANX1 may contribute to DMD pathogenesis, we first sought to establish whether PANX1 localisation or expression is altered in skeletal muscles from dystrophic patients. To this end, skeletal muscle biopsies collected from one donor without neuromuscular disease, one donor diagnosed with DMD, and one with an intermediate phenotype of DMD/BMD (complete absence of dystrophin staining on immunohistochemistry) were cross-sectioned and stained for PANX1. As presented in Supplemental Fig. 1, healthy skeletal muscle showed punctate and equally distributed PANX1 immunostaining, a pattern previously observed for Panx1 in this tissue [36, 38]. Conversely, the skeletal muscle sections from dystrophic patients displayed uneven staining throughout the tissue. These findings suggest that PANX1 distribution may be altered in skeletal muscles from dystrophic patients. Due to the difficulties in acquiring such samples, additional biological replicates could not be attained to confirm this. Thus, we then used immortalized human skeletal muscle myoblasts (HSMM) isolated from three healthy (Ctl 1–3) and 8 dystrophic donors (DMD 1–8) [31] and analyzed PANX1 levels via western blotting. Myoblasts from healthy donors displayed relatively similar PANX1 protein levels. Interestingly, myoblasts from dystrophic donors expressed varying levels of PANX1 (Fig. 1A-B). While PANX1 expression may be regulated differently from patient to patient, the mean PANX1 level in myoblasts from DMD patients was less than that of healthy donors.

Next, we wanted to determine if PANX1 channel activity is altered in myoblasts from dystrophic patients. To this end, PANX1 channel activity was assessed via sulforhodamine B (4 mg/ml) dye uptake, a dye permeable to PANX1 channels, after incubation in a either a low [K^+^] solution or the PANX1 activating high [K^+^] solution [41]. First, we confirmed the specificity of this assay by knocking down PANX1 levels using siRNA (*PANX1* siRNA) in healthy myoblasts. As shown in Fig. 1C, PANX1-knocked down myoblasts displayed a significant reduction in dye uptake in the presence of high [K^+^] compared to their control counterparts.

To represent the DMD group, we then selected dystrophic cell lines that express PANX1 levels that are lower (DMD 7), similar (DMD 1), and higher (DMD 2) than control myoblasts to assess PANX1 channel activity (3 Ctl and 3 DMD cell lines). While DMD 4 and DMD 6 cell lines also show low PANX1 protein expression, the DMD 7 line was selected as it was isolated from the patient with the highest blood creatine kinase levels at diagnosis (Supplementary Table 1 for available information on patients, modified from [31]). Creatine kinase in blood can be used as an indirect marker of muscle damage [4]. As anticipated, no dye uptake was observed in any Ctl or DMD cell lines after incubation in the low [K^+^] solution. Conversely, Ctl cells exposed to high [K^+^] displayed dye uptake incidences above 80% (Fig. 1D). Notably, all three dystrophic cell lines displayed a significantly lower incidence of dye uptake than that of the Ctl cells (Fig. 1D). Together these data suggest that despite varying PANX1 protein levels among dystrophic patient cell lines, PANX1 channel activity is consistently significantly impaired.

### Panx1^−/−^/mdx mice have a reduced life span, body weight, and lean mass

**Fig. 2:**
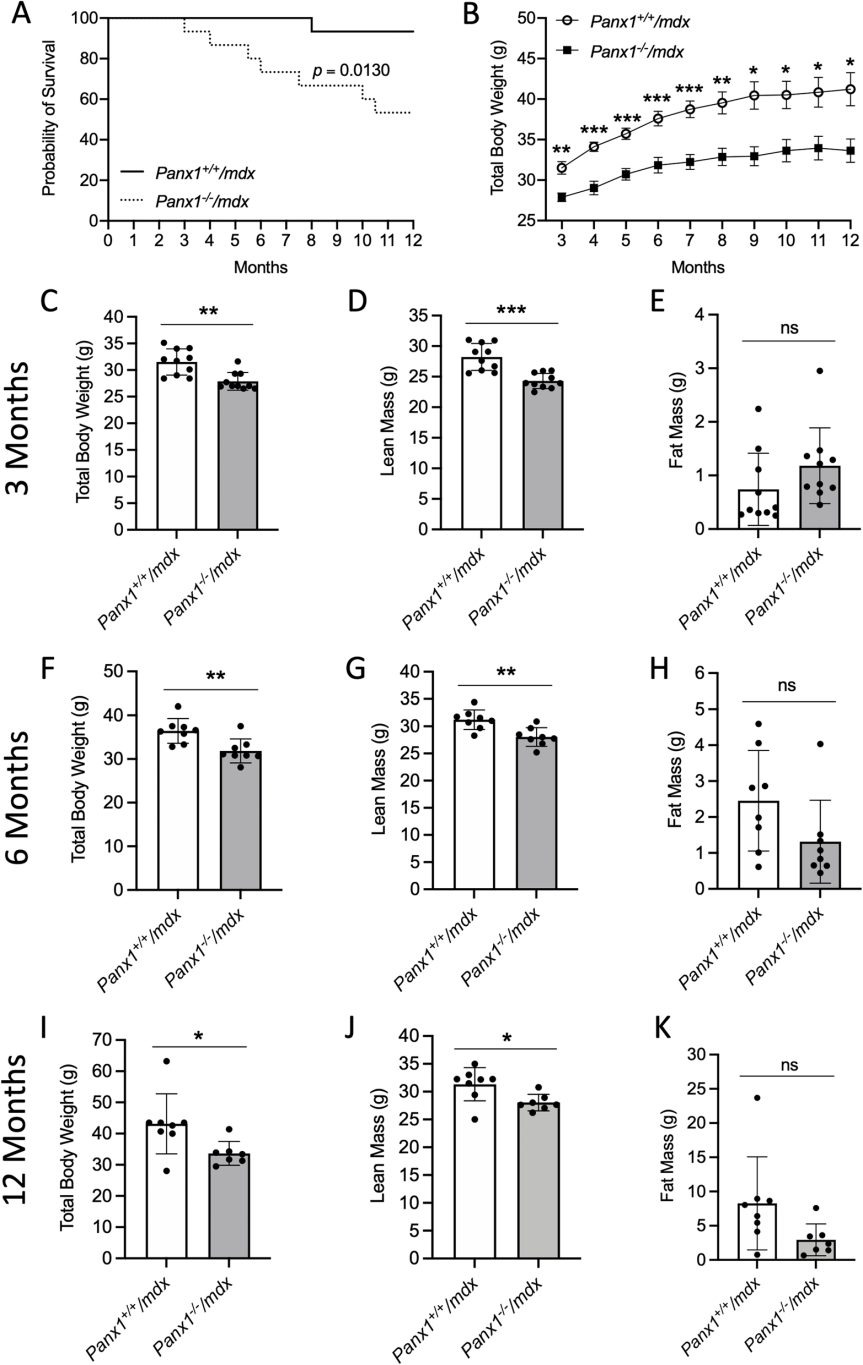
*Panx1*^*−/−*^*/mdx* mice Have a Reduced Life Span, Body Weight, and Lean Mass. **A** Survival curve of *Panx1*^*−/−*^*/mdx* and *Panx1*^+*/*+^*/mdx* mice (*n* = 15; Log-rank test). **B** Total body weight of *Panx1*^*−/−*^*/mdx* and *Panx1*^+*/*+^*/mdx* mice from 3–12 months of age (*n* = 8–15; two-tailed unpaired student’s *t*-tests for each time point). EchoMRI analysis displaying the **C**) total body weight, **D**) lean mass and **E**) fat mass of 3 month old mice (*n* = 10; two-tailed unpaired student’s *t*-test), **F**) total body weight, **G**) lean mass and **H**) fat mass respectively for 6 month old mice (*n* = 8; two-tailed unpaired student’s *t*-test) and **I**) total body weight, **J**) lean mass and **K**) fat mass respectively for 12 month old mice (*n* = 7–8; two-tailed unpaired student’s *t*-test). Data represents mean ± s.d., except for panel B that is shown as mean ± s.e.m. * P < 0.05, ** *P* < 0.01, *** *P* < 0.001, ns: non-significant

To study the role of Panx1 in dystrophic muscles, *Panx1*^*−/−*^ and *mdx* mice were crossed to generate *Panx1*^*−/−*^*/mdx* mice and their *Panx1*^+*/*+^*/mdx* controls. The *mdx* mouse is the most widely used preclinical model for DMD [14]. Given that Panx1 holds a predominant influence in male murine skeletal muscle [19] and since DMD primarily affects males [6, 23], only male mice were considered for this report. Notably, genetic ablation of *Panx1* in *mdx* mice was associated with a significantly reduced survival beginning as early as 3 months of age such that after 12 months the probability of survival in *Panx1*^*−/−*^*/mdx* mice decreased by ~ 50%. In contrast, the probability of survival in the *Panx1*^+*/*+^*/mdx* littermates only decreased by ~ 10% with a later onset at around 8 months of age (Fig. 2A). The total body weight of *Panx1*^*−/−*^*/mdx* mice was significantly lower than that of their *Panx1*^+*/*+^*/mdx* littermates (Fig. 2B).

To gain insight into the full body composition, *Panx1*^+*/*+^*/mdx* and *Panx1*^*−/−*^*/mdx* mice were subjected to EchoMRI analysis at 3, 6, and 12 months of age (Fig. 2C-K). In line with the above findings (Fig. 2B), 3-, 6- and 12-month-old *Panx1*^*−/−*^*/mdx* mice utilized for the EchoMRI had significantly lower body weight than that of *Panx1*^+*/*+^*/mdx* mice (Fig. 2C, F, I). Remarkably, the lean mass was significantly lower in *Panx1*^*−/−*^*/mdx* mice compared to their *Panx1*^+*/*+^*/mdx* counterparts (Fig. 2D, G, J) at all three ages, while there were no significant changes in the fat mass (Fig. 2E, H, K). These data are in line with findings from Oil Red O staining demonstrating no significant changes in the fat accumulation in either the TA or Sol of *Panx1*^*−/−*^* /mdx* mice and their *Panx1*^+*/*+^*/mdx* controls, which was found to be low in both genotypes (Supplemental Fig. 2). Together, these data indicate *Panx1*^*−/−*^*/mdx* mice have a shorter life span and a reduced body weight due to a lean mass deficit.

### The tibialis anterior muscles of Panx1^−/−^/mdx mice have a reduced mass and fiber number, together with an increased proportion of fibers with centrally located nuclei

**Fig. 3:**
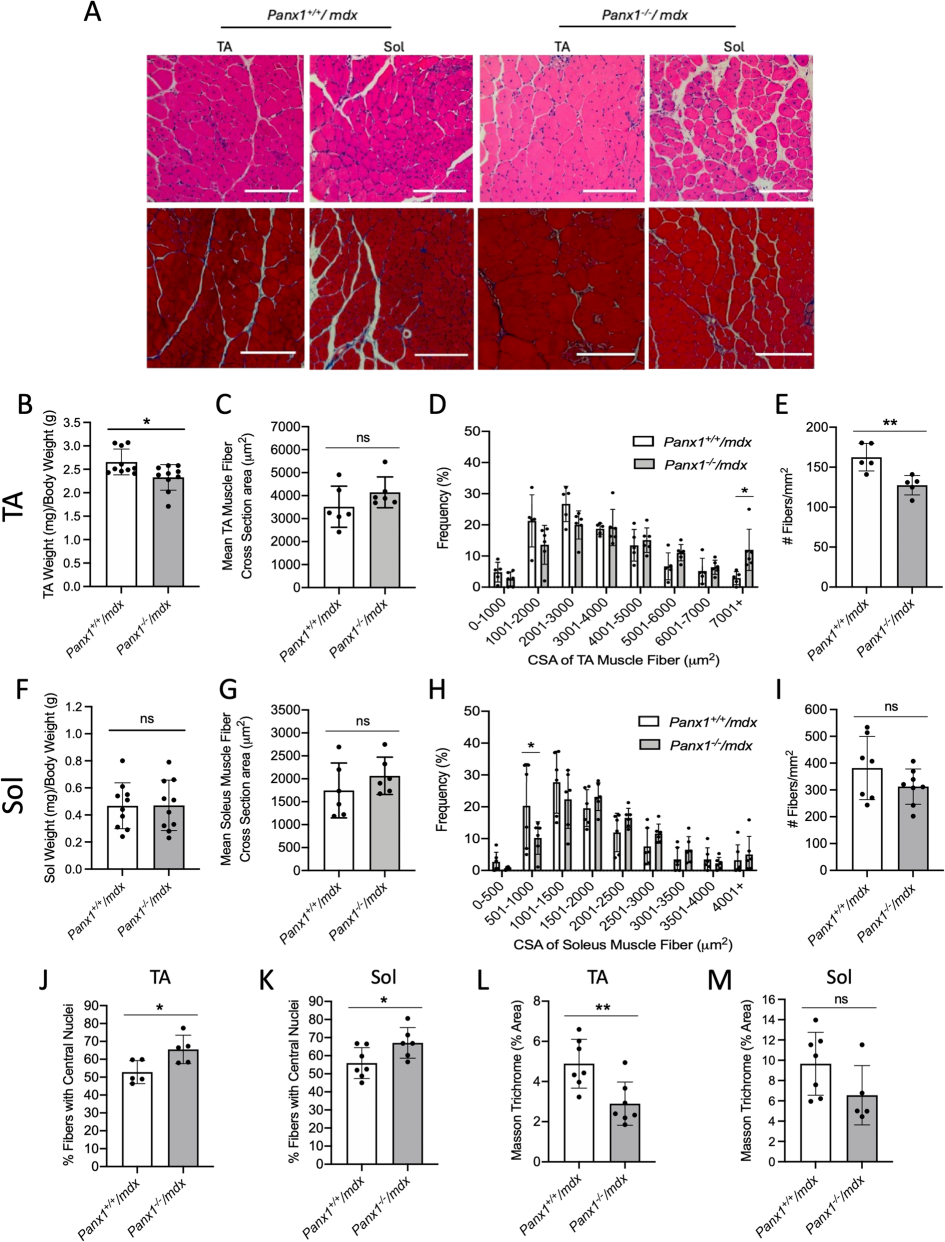
*Panx1*^*−/−*^*/mdx* mice TA Muscles are Smaller, Contain Less Myofibers with More that are Centrally Nucleated. **A** Representative images of the TA and Sol muscles of *Panx1*^*−/−*^*/mdx* and *Panx1*^+*/*+^*/mdx* cross-sections stained with H&E (top) and Masson trichrome (bottom). Scale bars = 200 μm. **B** The wet TA muscle weight was measured in both genotypes (*n* = 10; two-tailed unpaired student’s t-test). The TA **C**) mean CSA (*n* = 6; two-tailed unpaired student’s *t*-test), **D**) fiber CSA distribution (*n* = 6; two-way ANOVA followed by Sidak’s post hoc tests) and **E**) number of fibers per mm^2^ (*n* = 5; unpaired two-tailed student’s *t*-test) were measured. The Sol **F**) wet muscle weight (*n* = 10; unpaired two-tailed student’s t-test), **G**) mean CSA (*n* = 6; unpaired two-tailed student’s *t*-test), **H**) fiber CSA distribution (*n* = 6; two-way ANOVA followed by Sidak’s post hoc tests) and **I**) number of fibers per mm^2^ (*n* = 7–8; unpaired two-tailed student’s *t*-test) were also assessed. The percentage of fibers containing centrally located nuclei for **J**) the TA (*n* = 5; unpaired two-tailed student’s *t*-test) and **K**) the Sol (*n* = 6–7; two-tailed unpaired student’s *t*-test) were calculated. From the Masson trichrome staining the percentage of fibrotic area was calculated in the **L**) TA (*n* = 7; two-tailed unpaired student’s *t*-test) and the **M**) Sol (*n* = 5–7; two-tailed unpaired student’s *t*-test). Data represents mean ± s.d. * *P* < 0.05, ** *P* < 0.01, ns: non-significant

We next selected one fast-twitch and one slow-twitch muscle which we previously characterized in the *Panx1* knockout mouse model [19], namely the TA and Sol muscles respectively, for further investigation into the muscle histology of the *Panx1*^*−/−*^*/mdx* mice. The characterization of the two muscles is informative as fast-twitch (TA), but not slow-twitch (Sol), muscles in *mdx* mice have an increased susceptibility to contraction-induced injury [25]. These muscles were subjected to H&E staining for the analysis of fiber size, number, and presence of centrally located nuclei (Fig. 3A, top panels), and to Masson trichrome staining to analyze accumulation of fibrotic tissue (Fig. 3A, lower panels). The wet muscle masses were also recorded. The TA muscle weight of *Panx1*^*−/−*^*/mdx* (mean: 2.33 mg/g body weight) was significantly lower than that of their *Panx1*^+*/*+^*/mdx* counterparts (mean: 2.66 mg/g) (Fig. 3B). Interestingly, the average CSA of the TA in the *Panx1*^*−/−*^*/mdx* mice was not significantly different from that of *Panx1*^+*/*+^*/mdx* TAs however, the fiber size distribution showed a significantly increased proportion of fibers over 7000 µm2 (Fig. 3C-D). These data were accompanied by a significant drop in the number of muscle fibers per mm^2^ in the *Panx1*^*−/−*^*/mdx* TAs (Fig. 3E). In the Sol, no significant differences in muscle weight, fiber CSA or fiber number were observed between the two genotypes (Fig. 3F-I).

We next examined if loss of *Panx1* in dystrophic mice exacerbates the skeletal muscle’s pathology and susceptibility to damage. To this end, cross-sections of the TA and Sol were assessed for the percentage of fibers with centrally located nuclei, which represent myofibers undergoing regeneration following damage and also indicative of diseased muscle [8, 18]. Furthermore, sections were also subjected to Masson trichrome staining as an indicator of fibrosis (Van [45]). Both the TA and Sol muscles from *Panx1*^*−/−*^*/mdx* mice displayed significantly more fibers with centrally located nuclei compared to that of *Panx1*^+*/*+^*/mdx* mice (Fig. 3J-K). Intriguingly, the TA of *Panx1*^*−/−*^*/mdx* mice displayed less Masson trichrome staining than the *Panx1*^+*/*+^*/mdx* controls (Fig. 3L), but for both genotypes the muscle area stained with Masson trichrome was very low. No significant difference was observed for the Sol muscle (Fig. 3M). These data suggest that while loss of *Panx1* may slightly reduce the fibrotic accumulation in the TA of *mdx* mice, the TA muscles of *Panx1*^*−/−*^*/mdx* mice show enhanced muscle pathology with a significantly reduced mass and fiber number together with an enhanced proportion of fibers with centrally located nuclei.

### The tibialis anterior muscles of Panx1^−/−^/mdx mice have fewer SCs

**Fig. 4:**
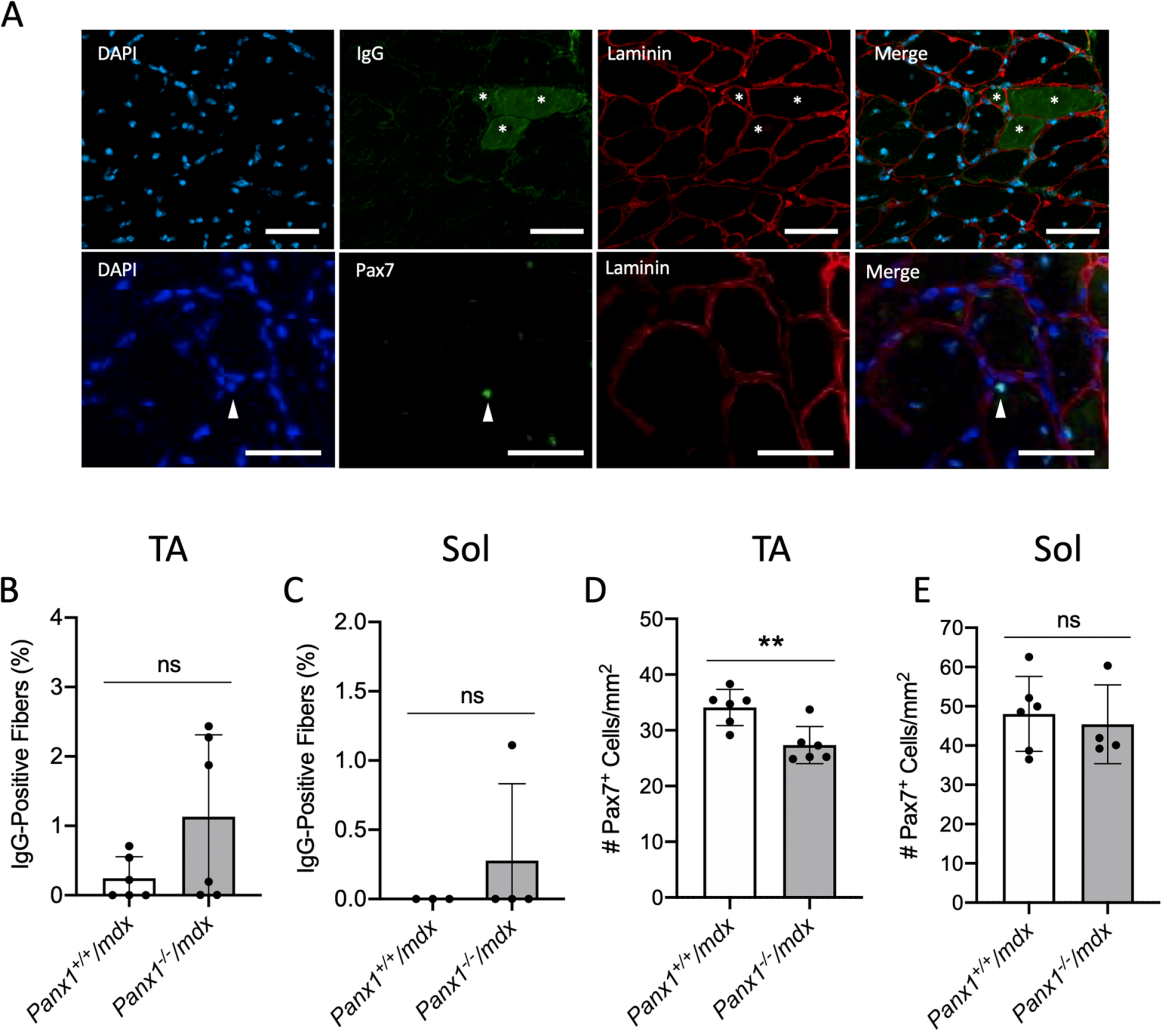
The *Tibialis Anterior* Muscles of *Panx1*^*−/−*^*/mdx* mice have Fewer SCs. **A** Representative pictures of cross-sections of the TA muscles of *Panx1*^*−/−*^*/mdx* mice stained for DAPI (blue), necrotic fibers (IgG positive; green) and laminin (red) across the top and stained for DAPI (blue), Pax7 (green) and laminin (red) across the bottom. Scale bar = 100 μm. Cross-sections were then analyzed for the percentage of IgG positive fibers for the **B**) TA (*n* = 6; two-tailed unpaired student’s *t*-test) and **C**) Sol (*n* = 3–4; two-tailed unpaired student’s *t*-test). The number of SCs per mm^2^, identified by Pax7 staining, were measured in D) TA (*n* = 6; two-tailed unpaired student’s *t*-test) and E) Sol muscles (*n* = 4–6; two-tailed unpaired student’s *t*-test). Data represents mean ± s.d. ** *P* < 0.01, ns: non-significant

We next sought to establish whether *Panx1*^*−/−*^*/mdx* had more necrotic fibers via IgG staining [5] (Fig. 4A, top panels). We also looked for any changes in the SC pool via Pax7 staining as loss of SCs can be associated with increased muscle damage [17] (Fig. 4A, bottom panels). While there was no statistical difference in the IgG-positive fibers between the two genotypes in either the TA (Fig. 4B) or Sol (Fig. 4C) muscles, overall the percentage of IgG-positive fibers were very low in both muscles suggesting that necrotic fibers have a minor contribution. Interestingly, while the SC numbers in the Sol muscle were comparable for both groups, genetic ablation of *Panx1* significantly decreased the SC number in the TA muscle of *mdx* mice (Fig. 4D-E).

### Panx1^−/−^/mdx mice display reduced muscle strength and locomotor function

**Fig. 5:**
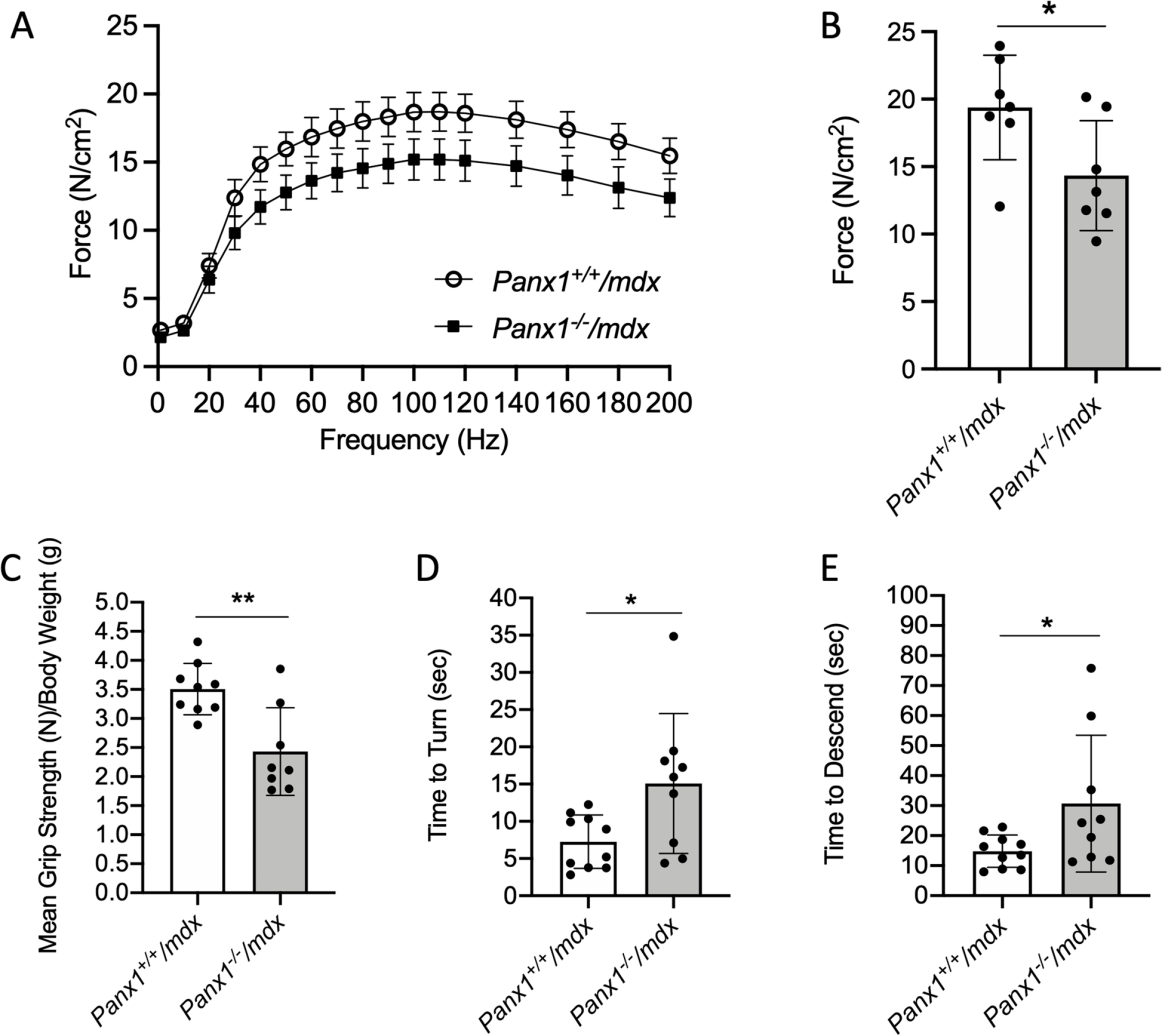
*Panx1*^*−/−*^*/mdx* mice Display Reduced Muscle Strength and Locomotor Function. The *soleus* muscle was subjected to force measurements and the **A**) force-frequency curves were recorded with the **B**) average maximum force determined based on the curves (*n* = 7; two-tailed unpaired student’s *t*-test). **C** Mean forelimb grip strength of *Panx1*^*−/−*^*/mdx* and *Panx1*^+*/*+^*/mdx* mice was measured (*n* = 8–9; two-tailed unpaired student’s *t*-test). Mice were also subjected to a pole test where the **D**) time to turn around and **E**) time to descend the pole were measured (*n* = 9–10; two-tailed unpaired student’s *t*-test). Data represents mean ± s.d. except for panel A where data represents mean ± s.e.m. to better visualize the curves. * *P* < 0.05, ** *P* < 0.01

As DMD is associated with muscle weakness, we then sought to determine whether *Panx1*^*−/−*^*/mdx* mice display reduced muscle strength. To this end, we first measured the maximum force of the Sol muscle. Due to its anatomical nature and the requirement of attaching the muscle tendon to tendon, the TA was not suitable for these tests. No shift was observed in the force frequency curve indicating that likely muscle fatigability was similar in the Sol muscles of the two groups of mice (Fig. 5A). Notably, when comparing the maximum force generated based on the curve, Sol muscles from *Panx1*^*−/−*^*/mdx* mice displayed a significant reduction in maximum force generation when compared to that of *Panx1*^+*/*+^*/mdx* (Fig. 5B).

Grip strength and pole tests were then used to further assess in vivo muscle strength and locomotor function. In line with the contraction data, *Panx1*^*−/−*^*/mdx* mice had significantly reduced grip strength than the *Panx1*^+*/*+^*/mdx* controls (Fig. 5C). When subjected to the pole test, *Panx1*^*−/−*^*/mdx* mice took a significantly longer time to turn around and descend the pole than the *Panx1*^+*/*+^*/mdx* mice (Fig. 5D-E). These findings taken alongside the reduced contractile force observed in the Sol muscle implicate that loss of Panx1 reduces muscle force and overall locomotor function.

### Increasing PANX1 levels promotes the differentiation and fusion of myoblasts from DMD patients

**Fig. 6:**
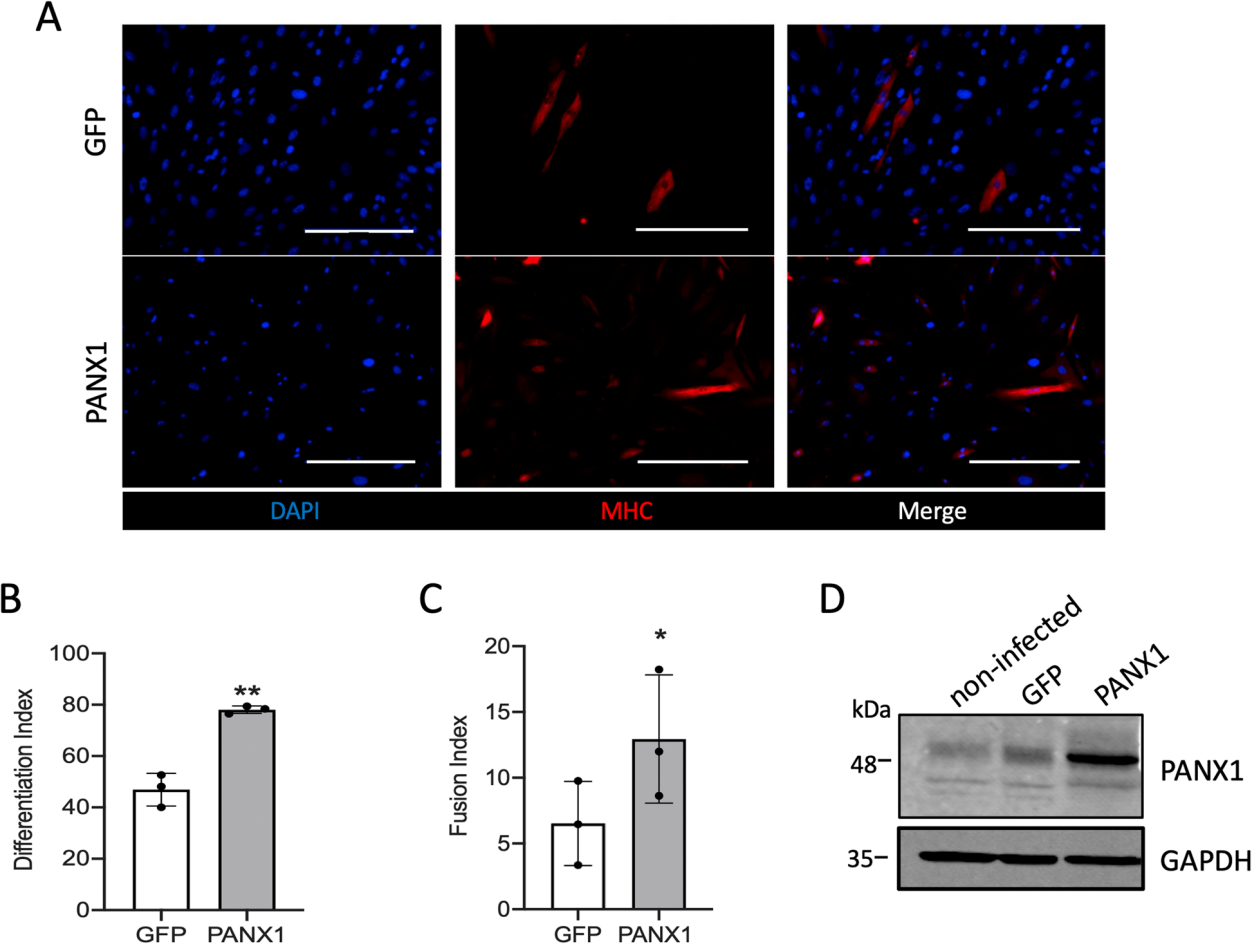
PANX1 Overexpression Increases the Differentiation and Fusion of Human Dystrophic Myoblasts. **A** Dystrophic human skeletal muscle myoblasts (HSMM) (namely DMD 1, DMD 2, and DMD 7) were transduced with a lentivector containing either a GFP or PANX1 overexpression construct. Cells were differentiated for five days then stained for DAPI (blue), and MHC (red). Representative images shown are from DMD 1. Scale bar = 400 μm. HSMM were then analyzed for the **B**) differentiation (*n* = 3; two-tailed paired student’s *t*-test) and **C**) fusion index (*n* = 3; two-tailed paired student’s *t*-test). Each symbol on the graphs represents one cell line. Data represents mean ± s.d. * *P* < 0.05, ** *P* < 0.01, ns: non-significant. **D** Western blot of PANX1 in DMD cells (DMD 7) non-infected or infected with a lentivector containing GFP or PANX1 overexpression construct. GAPDH was used as a loading control

Our current findings indicate that *Panx1* loss exacerbates several dystrophic features in *mdx* mice. As we have previously demonstrated that PANX1 overexpression in healthy HSMM cells promotes their differentiation and fusion in vitro [28], we wanted to assess whether increasing PANX1 levels can also promote muscle formation in the context of dystrophy. To this end, HSMM from DMD patients were transduced with either a PANX1 overexpression lentivector or a GFP lentivector as a control and induced to differentiate for five days. Myoblasts were then stained with the differentiation marker MHC (Fig. 6A) and the differentiation and fusion indices calculated. We again selected the DMD 1, DMD 2 and DMD 7 cell lines for this assay as they express PANX1 levels that are similar (DMD 1), higher (DMD 2), and lower (DMD 7) than control myoblasts. After five days of differentiation, dystrophic myoblasts overexpressing PANX1 displayed significantly higher differentiation (Fig. 6B) and fusion (Fig. 6C) indices compared to their counterparts overexpressing GFP (Fig. 6A-C). PANX1 overexpression was validated by western blotting (Fig. 6D). These data indicate that increasing PANX1 levels promotes the differentiation and fusion capacities of DMD myoblasts.

## Discussion

In this study, we demonstrate that while PANX1 levels varied amongst the dystrophic patient-derived myoblast cell lines assessed, its mean level and channel activity were significantly reduced compared to those originating from healthy donors. Our data suggests that the impairment of PANX1 channel activity is not directly or only correlated to its level in DMD cell lines. In normal skeletal muscle cells, the ATP released by Panx1 channels after electrical stimulation activates signaling pathways that regulate gene expression [7, 24]. Panx1 and dystrophin interact together in a multiprotein complex involved in this excitation-transcription coupling [3]. Due to absence of functional dystrophin, the assembly of this complex, subsequent ATP release, and signaling are likely disrupted in DMD. Indeed, Valladares et al. have measured the levels of extracellular ATP in isolated muscle fibers from C57BL/6 and *mdx* mice [44]. Basal levels of extracellular ATP were higher in dystrophic fibers but, as opposed to normal fibers, were unable to activate its release after electrical stimulation, suggesting an uncoupling between electrical stimulation and Panx1-mediated ATP release in dystrophic fibers. The dysregulation of PANX1 channel activity seen in DMD cell lines is thus likely primarily due to the absence of dystrophin and subsequent disruption of the multiprotein complex in which PANX1 and dystrophin participate in normal cells. This effect on PANX1 may be more pronounced in human muscle cells than in *mdx* mice as DMD patients are severely affected by the absence of dystrophin, while its consequence in *mdx* mice is milder [32]. However, in Becker muscular dystrophy (BMD) patients, in-frame mutations result in a truncated but partially functional dystrophin protein expressed at lower levels than in healthy muscle [20]. While the dystrophin domain involved in the multiprotein complex with Panx1 remains to be identified, truncated dystrophin expressed in BMD patients could potentially be part of the complex if the domain involved in this interaction is preserved.

In our previous studies, we had not observed a difference in Panx1 levels in the TA of 4-week-old *mdx* mice compared to age-matched C57BL/10ScSn control mice [36]. However, Cea et al. found an increase in Panx1 levels in the TA of 2- to 3-month-old *mdx*^*4c*^ mice compared to control C57BL/6 animals [9]. While both C57BL/10ScSn-*Dmd*^*mdx*/J^ (*mdx*) and *mdx*^*4cv*^ are considered mild models of DMD, the discrepancy in these findings could be due to differences in the mouse models used and their comparison control mice. It may also be related to their age as many pathologic features of the disease change throughout the first year of life. In C57BL/10ScSn-*Dmd*^*mdx*/J^ mice, disease features associated with cycles of muscle damage and repair start between 6 and 12 weeks of age [32] and thus not significant in the 4-week-old *mdx* mice used in our previous studies. On the other hand, it has been reported that the muscles of 3-month-old *mdx*^*4cv*^ mice display a dramatic increase in regenerating fibers compared to their age-matched controls [29]. We have previously shown that Panx1 levels increase in the TA muscle during regeneration following cardiotoxin-induced injury in the context of healthy mice [36]. It is thus possible that the increase in Panx1 levels seen in muscles of *mdx*^*4cv*^ mice at 2–3 months of age is due to the high cycling of muscle damage and repair. It is worth noting that *utrn*^*−/−*^*/mdx* mice show many more of the clinical signs of DMD than *mdx* mice [12]. Interestingly, and as stated above, Panx1 levels were significantly reduced in muscles from *utrn*^*−/−*^*/mdx* mice [36] which is in keeping with a reduction in the mean PANX1 level in myoblast cell lines from DMD patients shown here. While there are some discrepancies or differences amongst the data likely due to the various models (*mdx*, *mdx4*^*cv*^, *utrn*^*−/−*^*/mdx*), mice age, assays and muscle types used, all indicate that Panx1 levels and/or channel activity are altered in dystrophic mice.

To then examine the role of Panx1 in DMD, we generated a dystrophic (*mdx)* mouse model that lacks *Panx1* (*Panx1*^*−/−*^*/mdx*). We found that several dystrophic features were aggravated in these mice. Notably, the life span of *Panx1*^*−/−*^*/mdx* mice is significantly shorter than that of their control counterparts, which has not been observed in healthy *Panx1*^*−/−*^ mice. *Panx1*^*−/−*^*/mdx* mice also present with a significant loss of lean mass, potentially indicating muscle wasting. It is interesting to note that in the context of health, *Panx1* loss results in fat accumulation without altering body weight due to a concomitant reduction in lean mass [30], while lack of *Panx1* in dystrophic mice leads to a reduction in body weight due lean mass loss (no effect on fat mass). As it has been suggested that Panx1 channel activity is involved in the transcriptional changes linked to fast-to-slow muscle fiber phenotype transition [24], the dysregulation of Panx1 could make muscle fibers more permissive to damage. Indeed, slow-twitch dystrophic muscles are less susceptible to contraction-induced damage than fast-twitch dystrophic muscles [25, 35, 43]. Interestingly, we found that the fast-twitch TA muscle of *Panx1*^*−/−*^*/mdx* mice displayed altered fiber size distribution (higher proportion of myofibers over 7000 µm^2^), reduced wet weight, less myofibers, and fewer SCs. Conversely, the changes observed in the fiber size distribution (less myofibers between 501–1000 µm^2^ and reduction in fiber number in the Sol muscle were to a lesser degree than that of the TA and were not statistically significant. In addition, no changes in SC number or muscle weight were seen in the Sol muscle. These findings suggest that fast-twitch muscles such as the TA may be more susceptible to changes in Panx1 levels; an effect that was also observed in our previous study using global *Panx1* knockout mice [19]. We have previously shown that fibers from TA muscle of healthy male *Panx1*^*−/−*^ mice are smaller than control mice [19]. This was also observed in regenerating fibers following cardiotoxin induce injury, likely owing to impairment of myoblast fusion [19, 42]. As opposed to healthy mice, *mdx* mice present with myofiber hypertrophy mainly due to myofiber branching that continues throughout their lifespan. However, this is not reflected at the level of the whole muscle as a result of the concomitant myofiber loss [32]. Our findings thus suggest that in the context of dystrophy, the absence of *Panx1* leads to increased myofiber hypertrophy at least in the TA muscle of *mdx* mice, possibly because of myofiber branching, together with enhanced myofiber loss. This hypertrophy seemed insufficient to offset the myofiber loss as the TA muscle weight was less in *Panx1*^*−/−*^*/mdx* mice than in control animals. Histological analysis of the muscles from *Panx1*^*−/−*^*/mdx* mice further revealed increased signs of myofiber specific damage as seen by an elevated number with centrally located nuclei in both the TA and Sol muscles. The presence of myofibers with central nuclei is common to many neuromuscular disorders or diseases and has been suggested to contribute to muscle weakness and disease progression [18]. Interestingly, we found that despite an overall low amount of fibrosis in *Panx1*^+*/*+^*/mdx* skeletal muscle tissue, *Panx1*^*−/−*^*/mdx* muscles had slightly less. These data are in line with previous reports demonstrating that genetic ablation of *Panx1* reduces fibroblast activation and subsequently fibrosis in the liver [11] and cardiomyocytes [13]. Reduced fibrosis would typically be of benefit to dystrophic tissue. However, it did not seem to counteract the detrimental effects caused by the absence of Panx1 in *mdx* mice muscles as *Panx1*^*−/−*^*/mdx* mice presented with lower maximum force generation by the *soleus* muscle, reduced grip strength, and impaired locomotor function. These data further implicate a role for Panx1 in maintenance of muscle function and strength and that its dysregulation in DMD exacerbates disease progression, which is clearly indicated by the reduced lifespan of *Panx1*^*−/−*^*/mdx* mice.

Other organs may contribute to the reduced lifespan of *Panx1*^*−/−*^*/mdx* mice as Panx1 expression is not restricted to, and may affect [1], skeletal muscle. Respiratory and cardiac failure are the main causes of death in DMD patients and Panx1 is expressed in the heart [13] and lungs [37]. It has been previously reported that Panx1 levels are unaltered in the heart of *mdx* mice. Panx1 channel inhibition did not affect the extent of induced arrhythmia in mild (*mdx*) and severe (*utrn*^*−/−*^*/mdx*) DMD mouse models compared to untreated mice [22]. While Gonzalez et al. concluded that Panx1 does not contribute to DMD arrhythmogenesis, whether the reduced lifespan in *Panx1*^*−/−*^*/mdx* mice is due to cardio-respiratory failure remains to be examined. Future studies using skeletal muscle specific *Panx1* knockout mice will be beneficial to determine the contribution of skeletal muscle in the reduced lifespan observed here.

Since our data indicate that *Panx1* loss is detrimental to dystrophic skeletal muscles, we assessed whether increasing PANX1 levels could promote muscle formation in vitro using myoblasts from dystrophic patients*.* Similar to our previous findings obtained using primary skeletal muscle myoblasts from healthy patients [28, 36], PANX1 overexpression also increased the differentiation and fusion capacities of DMD patient-derived myoblasts. Based on this, it would be interesting to examine the potential benefit of increasing Panx1 levels in dystrophic mice in future studies. As PANX1 levels varied amongst the DMD cell lines, it would also be informative to determine whether PANX1 expression is correlated with dystrophy severity or other clinical features. However, the information available related to the patients (Supplemental Table 1) from which these cell lines were derived is insufficient to make such correlations. With an increased number of patients samples and more clinical information in future studies, we may be able to make correlations between PANX1 expression and features or severity of dystrophy. As our data suggests that PANX1 levels vary between fibers within dystrophic muscle specimens, correlation between the extent of morphometric abnormalities and PANX1 levels using serial sections, when possible, would also be beneficial.

Collectively, our findings suggest that PANX1/Panx1 dysregulation in DMD exacerbates several features of the disease such as muscle mass and myofiber loss, reduction of muscle strength and locomotion, and a significantly shortened lifespan. Moreover, our results suggest a potential therapeutic benefit to increasing PANX1 levels in dystrophic muscles to promote muscle formation and protect myofibers from the damage induced by the absence of dystrophin.

### Supplementary Information

**Supplementary Material 1.**

## Data Availability

The data that support the findings of this study are available from the corresponding author upon reasonable request.

## Abbreviations

ATP: Adenosine triphosphate
BMD: Becker Muscular Dystrophy
CSA: Cross-sectional area
Ctl: Control
DMD: Duchenne Muscular Dystrophy
EDL: Extensor digitorum longus
GA: Gastrocnemius
GFP: Green fluorescent protein
H&E: Hematoxylin and Eosin
HSMM: Human skeletal muscle myoblasts
MHC: Myosin heavy chain
PBS: Phosphate buffered saline
Panx1: Pannexin 1 (in reference to mouse)
PANX1: Pannexin 1 (in reference to human)
Quad: Quadriceps
SC(s): Satellite cells
siRNA: Short interfering ribonucleic acid
Sol: Soleus
TA: Tibialis anterior
