# Supplementary material for: Pannexin 1 dysregulation in Duchenne muscular dystrophy and its exacerbation of dystrophic features in mdx mice
Source: Skelet Muscle. 2024 Apr 26;14:8. doi: 10.1186/s13395-024-00340-8 (PMC11046831; doi:10.1186/s13395-024-00340-8)
Supplement: Supplementary file 1 — Supplementary Material 1. [file 13395_2024_340_MOESM1_ESM.pdf]

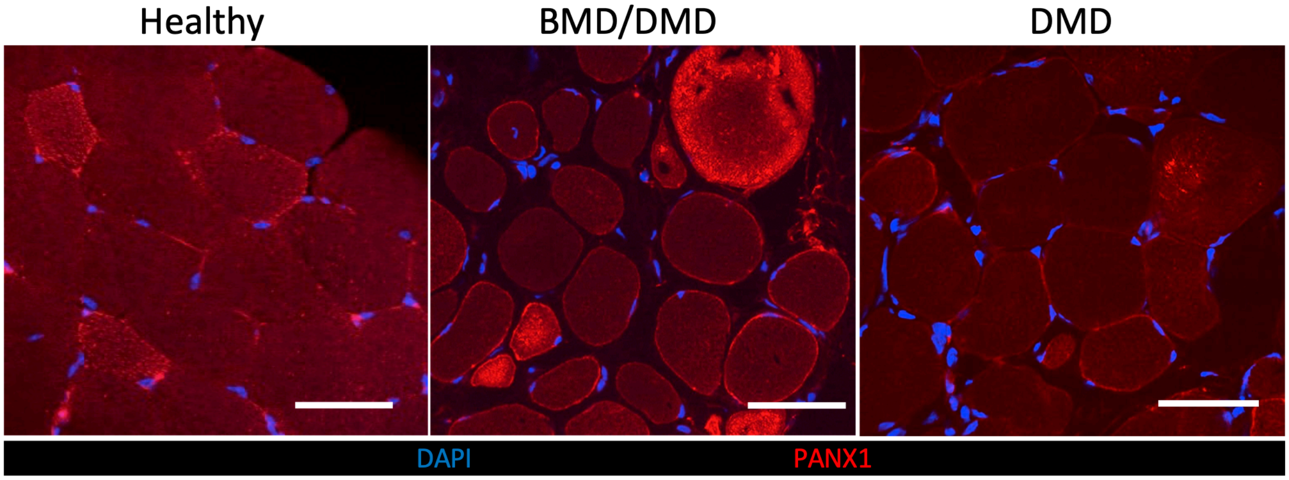

Supplemental Figure 1: PANX1 in muscle biopsy from a healthy donor and patients with dystrophy. Representative images of human biopsy samples collected from a healthy donor, a patient diagnosed with DMD, and one with BMD/DMD phenotype stained for PANX1 (red), and DAPI (blue). Scale bar = 50 μm.

Patient	Sex (M/F)	Age at Biopsy (m)	Diagnosis	Genetic Results	CK Blood Levels at Diagnosis (IU/L)	Age at First Walking (m)	Cardiomyopathy Age at Onset (m)	Scholiosis Age at Onset (m)	Scholiosis Surgery/Age at Surgery
Ctl 1	M	41	control	-	nl	24	N	N	N
Ctl 2	M	14	control	-	nl	24	N	N	N
Ctl 3	M	19	control	-	nl	18	N	N	N
DMD 1	M	54	DMD	c.3-26 duplication	18000	24	N	N	N
DMD 2	M	87	DMD	c.8-43 deletion	nd	13	Y/109	Y/141	Y/148
DMD 3	M	97	DMD	c.433 C>T substitution (p.R145X)	12881	15	N	Y/161	Y/161
DMD 4	M	25	DMD	C.8562 deletion A; pGlu2854Asp fs X2	19000	24	N	N	N
DMD 5	M	79	DMD	C.5758 C>T substitution; p.Gln1920X	8041	15	N	N	N
DMD 6	M	nd	DMD	Exon skipping 19 / IVS 19+1 G> C / c. 2380 + 1 G >C	nd	nd	Y/82	N	N
DMD 7	M	89	DMD	c.50-59 dup	47270	15	N	N	N
DMD 8	M	nd	DMD	nd	nd	nd	nd	nd	nd

Supplemental Table 1: Clinical data of patients from which immortalized myoblasts are derived. DMD, Duchenne muscular dystrophy; M, male; F, female; CK, creatine kinase; IU/L, international unit per liter; nl, normal; Y, yes; N, no; m, month; nd, non-determined. Table modified from Massenet et al., *Cells*, 2020, 9(8): 1780.

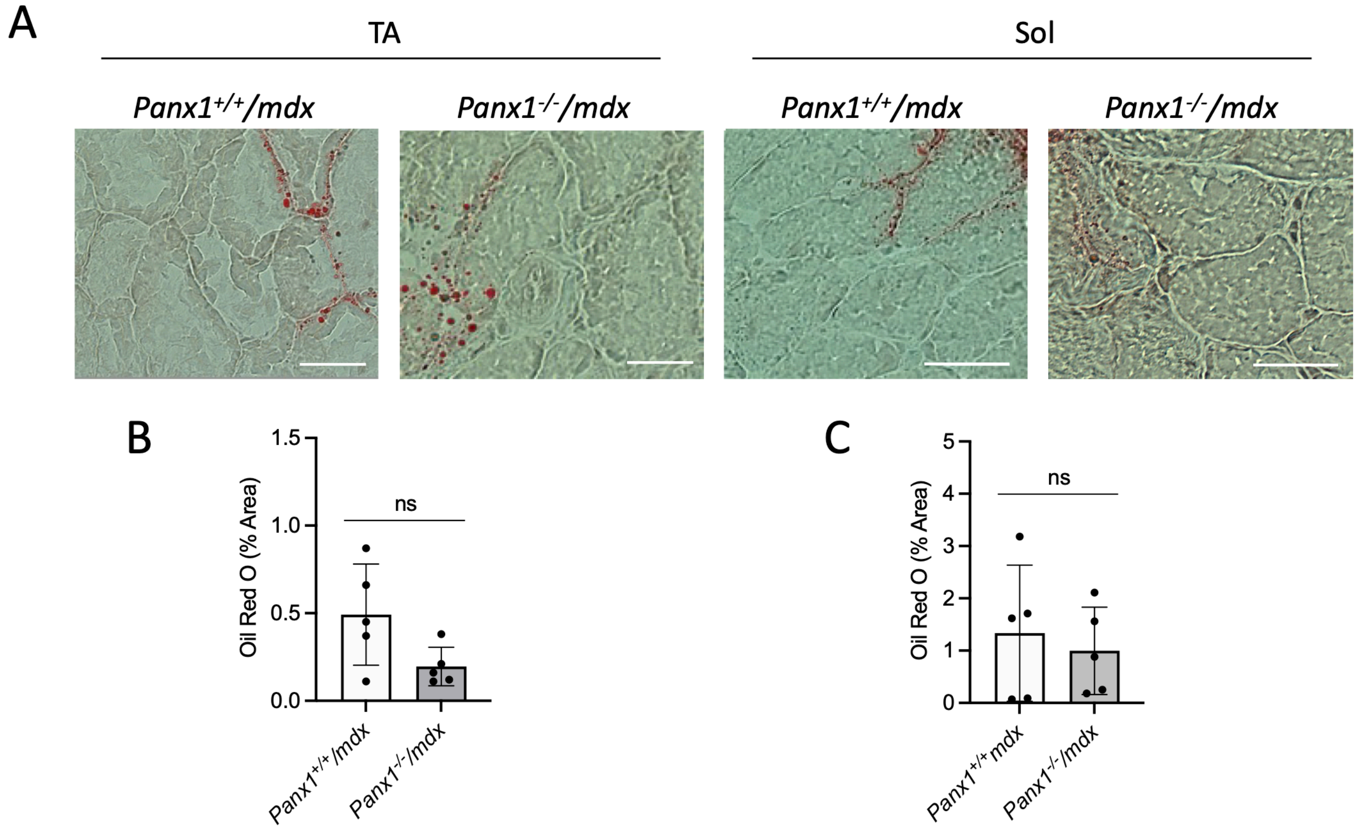

Supplemental Figure 2: TA and Sol muscles from *Panx1$^{-/-}$/mdx* mice do not display enhanced fat infiltration. Representative images of cross-sections of the TA and Sol muscles of *Panx1$^{-/-}$/mdx* and *Panx1$^{+/+}$/mdx* mice stained with Oil Red O. Scale bar = 50 μm. The percentage of Oil Red O positive area was calculated in the B) TA (n=5; two-tailed unpaired student's *t*-test) and the C) Sol (n=5; two-tailed unpaired student's *t*-test). Data represents mean ± s.d. ns: non-significant.

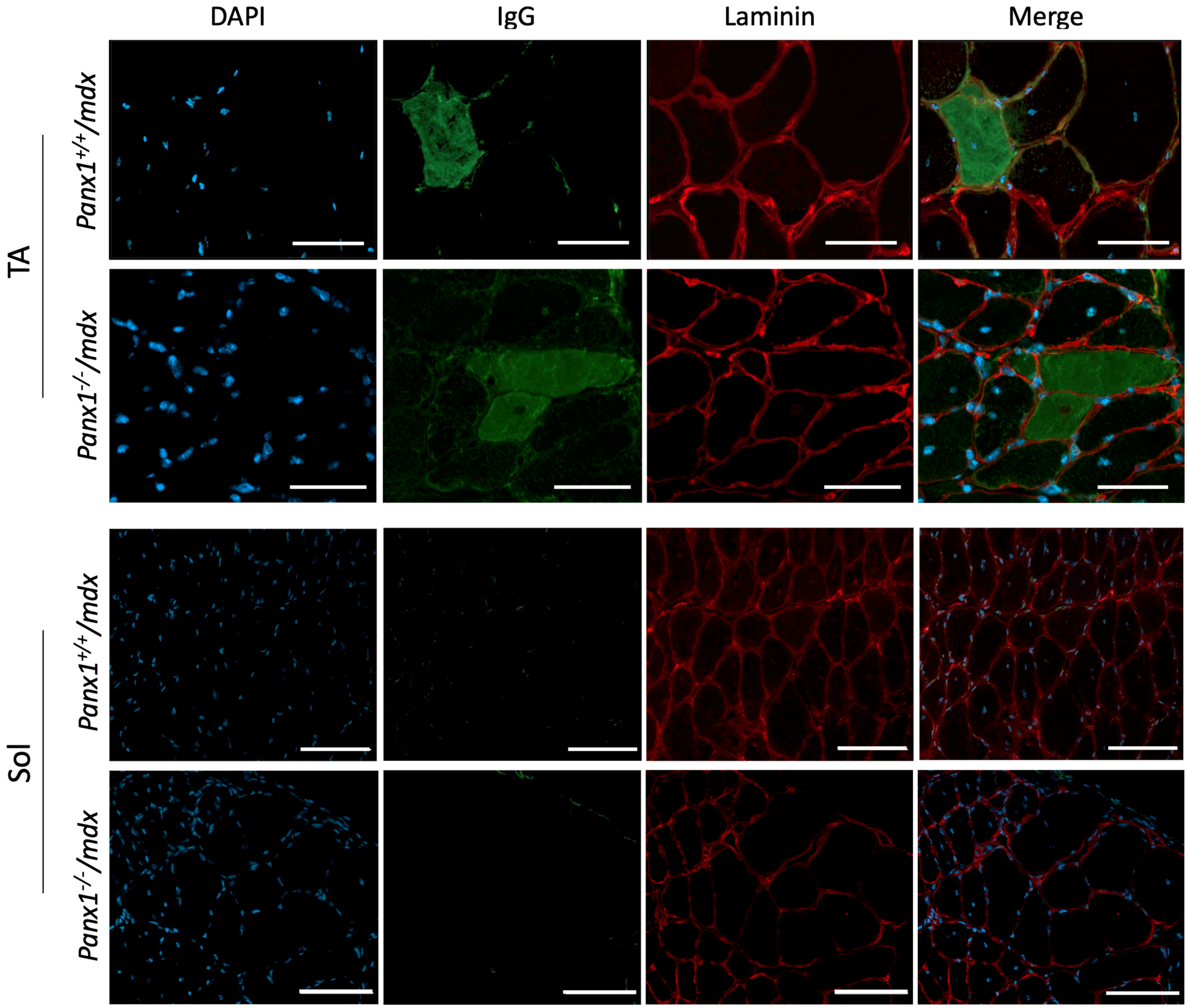

Supplemental Figure 3: Representative images of TA and Sol muscle cross-sections of *Panx1$^{-/-}$/mdx* and *Panx1$^{+/+}$/mdx* mice stained for necrotic fibers. Representative pictures of cross-sections of the TA and Sol muscles of *Panx1$^{-/-}$/mdx* and *Panx1$^{+/+}$/mdx* mice stained for DAPI (blue), necrotic fibers (IgG positive; green) and laminin (red). Scale bar = 100 µm.

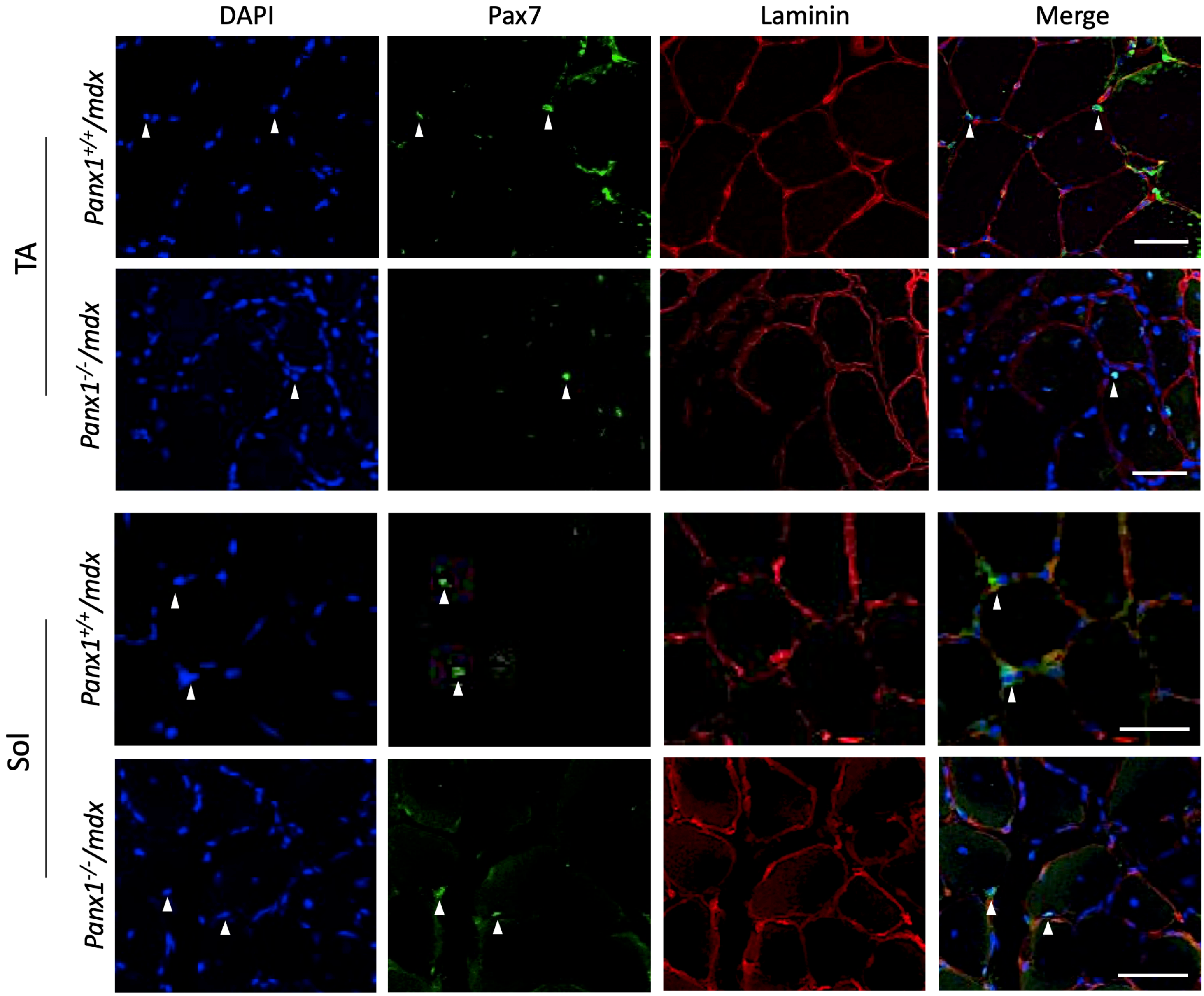

Supplemental Figure 4: Representative images of TA and Sol muscle cross-sections of *Panx1$^{-/-}$/mdx* and *Panx1$^{+/+}$/mdx* mice stained for Pax7 and laminin. Representative pictures of cross-sections of the TA and Sol muscles of *Panx1$^{-/-}$/mdx* and *Panx1$^{+/+}$/mdx* mice stained for DAPI (blue), satellite cells (Pax7 positive; green) and laminin (red). Scale bar = 50 μm.
